# Outdoor Performance Analysis of a Photovoltaic Thermal (PVT) Collector with Jet Impingement and Compound Parabolic Concentrator (CPC)

**DOI:** 10.3390/ma10080888

**Published:** 2017-08-01

**Authors:** Ahed Hameed Jaaz, Husam Abdulrasool Hasan, Kamaruzzaman Sopian, Abdul Amir H. Kadhum, Tayser Sumer Gaaz, Ahmed A. Al-Amiery

**Affiliations:** 1 Solar Energy Research Institute, Universiti Kebangsaan Malaysia, Bangi, Selangor 43600, Malaysia; hussam2003hussam@yahoo.com; 2 Department of Chemical & Process Engineering, Faculty of Engineering & Built Environment, Universiti Kebangsaan Malaysia, Bangi, Selangor 43600, Malaysia; amir@eng.ukm.my; 3 Department of Mechanical & Materials Engineering, Faculty of Engineering & Built Environment, University Kebangsaan Malaysia, Bangi, Selangor 43600, Malaysia; taysersumer@gmail.com; 4 Department of Machinery Equipment Engineering Techniques, Technical College Al-Musaib, Al-Furat Al-Awsat Technical University, Al-Musaib, Babil 51009, Iraq; 5 Energy and Renewable Energies Technology Centre, University of Technology, Baghdad 10001, Iraq; dr.ahmed1975@gmail.com

**Keywords:** photovoltaic thermal collectors, water based PVT, electrical performance, thermal performance, compound parabolic concentrator, jet impingement

## Abstract

This paper discusses the effect of jet impingement of water on a photovoltaic thermal (PVT) collector and compound parabolic concentrators (CPC) on electrical efficiency, thermal efficiency and power production of a PVT system. A prototype of a PVT solar water collector installed with a jet impingement and CPC has been designed, fabricated and experimentally investigated. The efficiency of the system can be improved by using jet impingement of water to decrease the temperature of the solar cells. The electrical efficiency and power output are directly correlated with the mass flow rate. The results show that electrical efficiency was improved by 7% when using CPC and jet impingement cooling in a PVT solar collector at 1:00 p.m. (solar irradiance of 1050 W/m^2^ and an ambient temperature of 33.5 °C). It can also be seen that the power output improved by 36% when using jet impingement cooling with CPC, and 20% without CPC in the photovoltaic (PV) module at 1:30 p.m. The short-circuit current *I_SC_* of the PV module experienced an improvement of ~28% when using jet impingement cooling with CPC, and 11.7% without CPC. The output of the PV module was enhanced by 31% when using jet impingement cooling with CPC, and 16% without CPC.

## 1. Introduction

**Figure 1:**
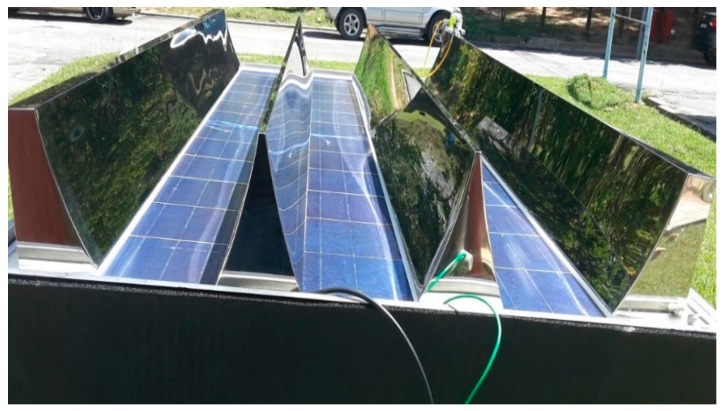
Photograph of the photovoltaic compound parabolic concentrator (PVT-CPC) collector.

Solar energy is a provider of clean and green energy, which can be used to fulfill global energy needs. A hybrid photovoltaic thermal (PVT) system changes solar energy to thermal energy, while a photovoltaic solar cell changes it to electrical energy [1]. The aforementioned system is the result of a combination of solar cells and a thermal collector, which changes sunlight into electricity while removing any remaining heat from the PV module. The electrical efficiency of photovoltaic solar cells is inversely proportional to the temperature; this is attributed to the inherent increase in the resistance of the system. There are many systems that can be designed and equipped to remove extra heat from the PV cells as a manner of cooling, which helps enhance its efficiency due to its lowered resistance. A solar collector is crucial in the solar energy system, as it changes solar radiation into heat for the working fluids. PV solar cells convert solar radiation into electricity. In normal cases, these two systems are entirely separate. However, both can be merged to form a hybrid PVT system. The hybrid system has been extensively studied since the 1970s. Most PVT collectors use water or air to cool solar cells or transfer heat to the working fluid. Huang et al. [2] proposed improvements that would result in increasing PV efficiency by 9%, thermal efficiency by 44.5% and total efficiency by 53.5% for the PVT collector. He et al. [3] realized a PV efficiency of 5.42%, a thermal efficiency of 51.94% and a total efficiency of 57.38%. Zondag et al. [4] analyzed multiple concepts of combined PV-thermal collectors. A total of nine designs were analyzed to determine the one with the highest efficiency. The channel-below-transparent-PV design reported a PV efficiency of 9%, a thermal efficiency of 63% and a total efficiency (PVT efficiency) of 72%, which is the best in this case. Chow et al. [5] studied the influence of glass cover on both the energy and exergy of a PVT collector. The experimental results show that the thermal energy for the unglazed PVT system exceeds that of the glazed PVT collector. It was also observed that the efficiency of the PV cell in the case of the unglazed PVT system exceeds that of the glazed PVT collector. Kong et al. [6] analyzed the effect of the Fresnel lens and two flat mirrors (solar connector) on both the electrical and thermal efficiencies of a low concentrated photovoltaic thermal system (CPVT). A total of 18 photovoltaic solar cells were installed on the aluminum receiver. On a clear day, it was reported that the electrical and thermal efficiencies increased by 10% and 56%, respectively. Li et al. [7] experimentally analyzed the performance based on the trough concentrating photovoltaic thermal (TCPVT) system of solar cell arrays at multiple irradiance intensities. It was proven that the GaAs solar cells performed well under concentrated conditions. Dupeyrat et al. [8] analyzed a single glazed flat plate photovoltaic thermal water collector. He reported that the standard PV panel resulted in increased thermal efficiency and decreased electrical efficiency, which is attributed to glazing. Calise et al. [9] concluded that enhanced beam radiation increases the efficiency of the manifolds. Bahaidarah et al. [10] reported that the power output of the photovoltaic compound parabolic concentrator (PV-CPC) system is higher than flat PVs. The power output for the PV-CPC system installed with a cooling system was 39% higher, 23% more than the flat PV system. Abu-Bakar et al. [11] analyzed the influence of an asymmetrical compound parabolic concentrator on both thermal and electrical performance, and reported that coupling the PVT system with a concentrating photovoltaic system results in increased electrical output of the system. Fujisawa and Tani [12] compared the annual performance of a single glazed PVT collector with monocrystalline silicon solar cells, a flat plate solar water heating collector (SWC), a PV module and an unglazed PVT collector. In the context of energy gain, the single glazed PVT collector was the best, followed by the SWC, the unglazed PVT and the PV module, while in the context of exergy analysis, the unglazed PVT was the best, followed by the PV module, the single glazed PVT and the SWC. Tripanagnostopoulos et al. [13] compared the glazed and unglazed PVT systems for the water heating collector, and reported that the former improved the thermal efficiency by ~30% and decreased the electrical efficiency by ~16% as opposed to the unglazed systems. Coventry and Lovegrove [14] numerically investigated the performance of the water-type PVT system, while Bosanac et al. [15] reported a decrease of 28% and 6% in the power output of the glazed and unglazed PVT system, respectively. Garg and Adhikari [16] pointed out that both systems were superior to the compound parabolic concentrator (CPC). Coventry [17] observed that the thermal and electrical efficiency of a PVT solar collector were ~58% and ~11%, respectively. Othman et al. [18] outlined that the efficiency of a PVT solar air collector exhibited significant improvement due to the utilization of CPC and fins. Tchinda [19] stipulated that the outlet temperature of air is inversely related to the mass flow rate of air. Guiqiang et al. [20] confirmed that the efficiency of PV cells is enhanced due to the utilization of CPC in buildings that are installed with a PVT collector. Atheaya et al. [21] investigated the characteristic equation for partially covered PVT-CPC. He proved that the partially covered PVT-CPC water collector system with a 25% PV module performed better than the others. It should also be pointed out that the fully covered PVT-CPC water collector system is capable of meeting the electrical and thermal demands. The electrical efficiency of the partially covered PVT-CPC water collector system is inversely correlated with the solar cell temperature. We designed a photovoltaic thermal water collector equipped with a CPC shown in Figure 1. The back of the PV cell is cooled by a jet impingent of water, while the CPC refocus and redirect solar radiation onto the PV cells. The aforementioned jet of water enhances heat transfer to the water, which subsequently increases the electrical and thermal performance of the system. The current PVT with jet impingement design system was evaluated in order to determine the efficiencies of the PV module and thermal system. Table 1 summarizes a comparison of the PVT collector between the current study and other absorber collector designs. In this work, the jet impingement will be used to reduce the temperature of the PV module while using the CPC to increase the solar intensity for the PV module.

## 2. Materials and Method

The water based PVT solar collector system of 1490 mm × 975 mm × 50 mm installed with a jet impingement of a 6 mm diameter stainless steel tube and CPC is shown in Figure 1. The PVT solar collector system has, at a minimum, one inlet and outlet to allow the medium (water) to enter and exit from the PVT solar collector system, respectively. The jet impingement cooling system was designed and configured with 36 nozzles in order to direct jet water to the back of the PV module. The hot water is collected in a storage tank. In the current experiment, the PV module was tested. The jet impingement cooling system was placed on the back of the PV module. A K-type thermocouple was used to measure the ambient and other temperatures. The thermocouple is located at multiple places and connected directly to a data logger. A Kipp and Zonen Pyranometer (Delftechpark, Delft, Netherlands) was fixed on the PVT collector to measure solar irradiance from the sun. The mass flow rate of jet impingement varied from 0.083 to 0.25 kg/s. The ADAM Data Acquisition System (5750 SW, Beaverton, OR, USA) was used to collect data, which was then stored in a computer at every minute. These data will later be used to determine the performance of the system.

## 3. Experimental Set-Up

**Figure 2:**
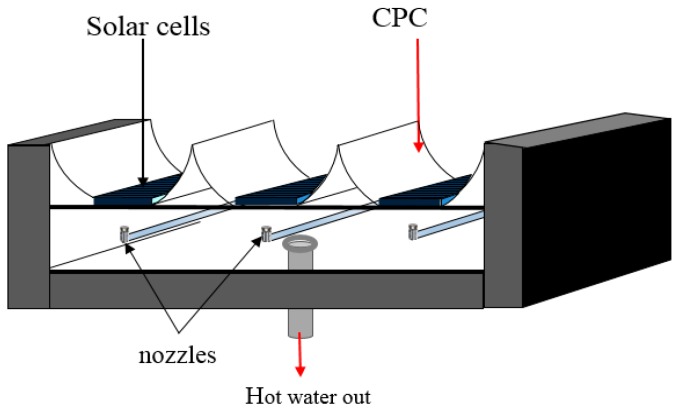
The schematic diagram of the PVT-CPC collector.

**Figure 3:**
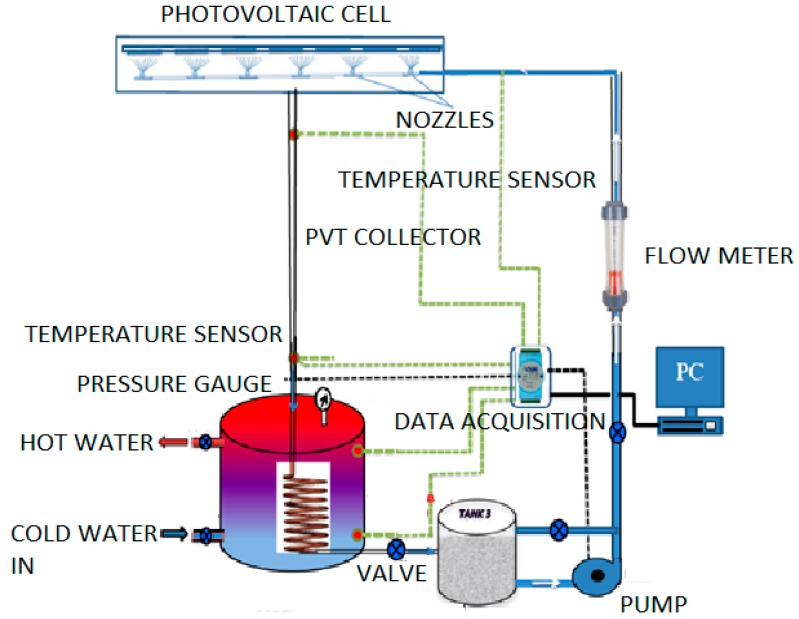
The schematic diagram of the PVT collector with compound parabolic concentrators.

**Figure 4:**
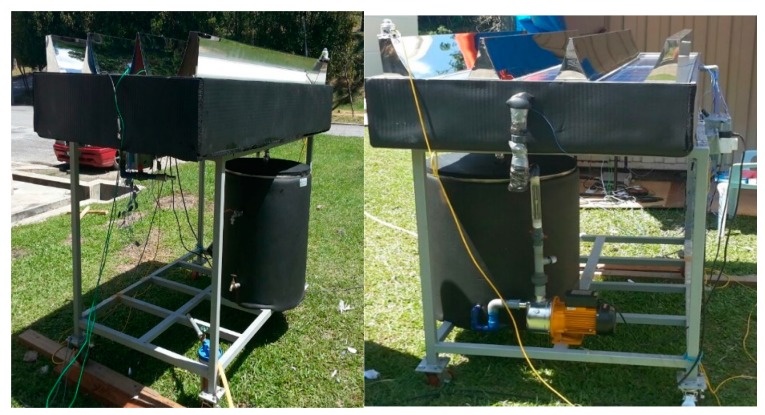
Photograph of the experimental set-up.

The PVT water collector was tested at the Solar Energy Research Institute (SERI), Universiti Kebangsaan Malaysia. The control parameters (indoor test) include the PV mean, input, output and ambient temperatures, wind velocity at the collector surface, useful current and voltage and water jet to the PV module. These parameters match the standards set for the PVT absorber collector. The construction of the photovoltaic module and CPC display are shown in Figure 1. In this study, the PV module was constructed from 36 thin wafers of polycrystalline silicon, measuring 156 mm by 156 mm, and 200 microns thick. Table 2 tabulates the electrical characteristics of the polycrystalline silicon photovoltaic module. The jet impingement takes place at the back of the PV module, as shown in Figure 2. A total of 36 nozzles were used for jetting water to cool the back of the PV module. PVT water collectors were installed with the jet impingement system and tested in a laboratory at multiple mass flow rates of water jet impingement. The testing procedure of the PVT water collector standard was used to test the novel design of the PVT solar collectors installed with the jet impingement system. The experimental set-up and complete measuring system for the PVT collector are shown in Figure 3 and Figure 4, respectively. The experimental testing was conducted under steady-state conditions to determine the performance of the PVT system. The thermal performance of the PVT collectors can be tested by obtaining the instantaneous efficiencies of different combinations of incident solar radiation, inlet fluid temperature, outlet fluid temperature and ambient temperature. The PVT collector with the jet impingement system was tested outdoors from 9:00 a.m. until 4:00 p.m. The effect of mass flow rates of 0.083, 0.166, 0.25 and 0.333 kg/s were duly tested. The thermocouple was used to determine the temperatures at several points in the PVT collectors. A total of 18 thermocouples were uniformly fixed to the back of the PV module to measure the mean PV temperature. Other thermocouples were fixed on top of the PV module, water tank, inlet and outlet fluid, and at the base of the PVT collector. A data acquisition system with 32 channels was connected to the computer system to record the data from the PVT collector, and stored every minute. The data can be used to calculate the electrical, thermal and PVT efficiencies of the PVT collector for changing mass flow rate and solar irradiance levels. A water pump was used to activate the jet water to cool the PV module, while hot water was collected in the thermal collector and connected to the heat exchanger, then channeled to the storage tank to form the closed-loop system shown in Figure 4. 

## 4. Energy Analysis

The thermal efficiency ($\eta_{th}$) and electrical efficiency ($\eta_{ele}$) for the system were duly determined, due to the fact that both are representative of the system’s performance. The analytical parameters of the PVT collector are tabulated in Table 3.

The performance of the system is represented by Equation (1) [22]:

(1) $$\eta_{PVT}\;=\;\eta_{elc}+\eta_{th}$$

Its performance is reliant upon many parameters. In the current study, the PVT system was analyzed using multiple mass flow rates. The PVT collector was assumed to be a flat-plate collector with a single glazing sheet. This assumption allowed us to utilize the Hottel–Whillier equations to study the thermal performance of the PVT collector [22]. The thermal efficiency of a conventional flat-plate solar collector is the ratio of the useful thermal energy ($Q_{u}$) to the solar Irradiance (I), expressed by:

(2) $$\eta_{th}\;=\;\frac{Q_{u}}{I}$$

The useful gain heat collected by the flat-plate solar collector can represent the combination of the average mass flow rate ($\dot{m}$), heat capacity of flowing medium ($C_{P}$), and temperature difference at the collector inlet ($T_{i}$) and outlet ($T_{o}$):

(3) $$Q_{u}\;=\;\dot{m}C_{P}\left(T_{o}-T_{i}\right)\;$$

The Hottel–Whillier (Equation (4)) defines the difference between the absorber solar radiation and thermal heat losses [22]:

(4) $$Q_{u}=A_{c}F_{R}\left[G_{T}{\left(\tau \alpha \right)}_{PV}-U_{L}\left(T_{i}-T_{a}\right)\right]$$

where $A_{c}$ is the collector area, $T_{a}$ is the ambient temperature, $T_{i}$ is the inlet temperature, $U_{L}$ is the overall collector heat loss, $\eta_{th}$ is the PV thermal efficiency, $G_{T}$ is the solar radiation at NOCT (radiation level 800 W/m^2^, wind velocity 1 m/s and ambient temperature 26 ℃), and $F_{R}$ is the heat removal efficiency factor introduced. This factor is expressed as follows:

(5) $$F_{R}=\frac{\dot{m}\;C_{p}}{A_{C}\;U_{L}}\left[1-\exp\left(-\frac{A_{C}U_{L}F^{\prime }}{\dot{m}{\;C}_{p}}\right)\right]$$

where $F^{\prime }$ is a constant which refers to the collector efficiency factor. 

The overall loss coefficient ($U_{L}$) of the collector is the sum of the edge ($U_{e}$) and top ($U_{t}$) loss coefficients, and can be expressed [22] as:

(6) $$U_{L}=\;U_{e}+\;U_{t}$$

(7) $$U_{e}=\frac{k_{e}p\;l}{L_{c}A_{c}}$$

(8) $$U_{t}\;=\left[{\left\{\frac{N}{\frac{c}{T_{\mathrm{pm}}}{\left[\frac{T_{\mathrm{pm}}\;-\;T_{a}}{\left(N\;+\;f\right)}\right]}^{e}}\;\frac{1}{h_{w}}\right\}}^{-1}+\;\frac{\sigma \left(T_{pm}\;+\;T_{a}\right)\left(T^{2}{}_{\mathrm{pm}}\;+\;T^{2}{}_{a}\right)}{{\left(\varepsilon +0.00591\;N\;h_{w}\right)}^{-1}\;+\;\frac{2N+f-1+0.133\varepsilon_{P}}{\varepsilon_{g}}\;-\;N}\right]\;$$

where,

(9) $$C=\;520(1-0.000051\beta^{2})$$

(10) $$f=\;\left(1+0.089h_{w}-0.1166h_{w}\varepsilon_{p}\right)\left(1+0.07866N\right)$$

(11) $$e=0.43\left(1-\frac{100}{T_{\mathrm{pm}}}\right)$$

(12) $$T_{\mathrm{pm}}=T_{i}+\frac{\frac{Q}{A_{c}}}{F_{R}U_{L}\;}\left(1-F_{R}\right)$$

(13) $$T_{pm}=\frac{T_{up}\;+\;T_{bm}}{2}\;$$

$T_{pm}:$ Mean temperature of PV module
$T_{up}$: Top plate temperature

(14) $$T_{bm}=\frac{\left(T_{1}+\;T_{1}+T_{3}+T_{4}+T_{5}+T_{6}+T_{7}+\ldots +T_{18}\right)}{18}$$

where $T_{bm}$ means the temperature of the back PV module, N  is the number of glass covers, σ is the Stefan-Boltzmann constant, $\varepsilon_{p}\;$ is the plate emittance, $\varepsilon_{G}\;$ is the glass emittance, β is the collector tilt, $T_{pm}$ is the mean plate temperature and $h_{w}$ is the wind heat transfer coefficient. The heat transfer coefficients can be calculated using Equation (15), while the natural heat transfer coefficient ($h_{nat}$) can be calculated using Equation (16) [22], as follows:

(15) $$h_{w}=2.8+3.0v$$

(16) $$h_{nat}=1.78\left(T_{pm}-T_{a}\right)$$

A combination of the natural and forced convection heat transfer coefficients (Equations (15) and (16)) determines the overall convection heat transfer ($h_{c}$) and possibly the overall top loss heat transfer coefficient for the collector [22].

(17) $$h_{c}=\sqrt[{\;}]{h_{w}{}^{3}+h_{nat}{}^{3}}$$

Equations (3)–(5) can be used to determine the useful heat gain emitted by the PVT collector. The reorientation of Equation (3) can be used to determine the thermal efficiency of the collector [22]:

(18) $$\eta_{th}=F_{R}\left(\tau \alpha \right)-F_{R}U_{L}{}_{\;}\left(\frac{T_{i}\;-\;T_{a}}{G_{T}}\right)$$

The electrical efficiency of the PV module ($\eta_{ele}$), which is a function of module temperature, is given by [1]:

(19) $$\eta_{ele}=\eta_{r}\left(1-\;\gamma \left(T_{c}-T_{r}\right)\right)$$

where $\eta_{r}\;$ is the reference efficiency of the PV module ($\eta_{r}\;$ = 0.12),  γ is a temperature coefficient (γ = 0.0045℃), Tc is the cell temperature and $T_{r}$ is the reference temperature.

## 5. Results and Observations

**Figure 5:**
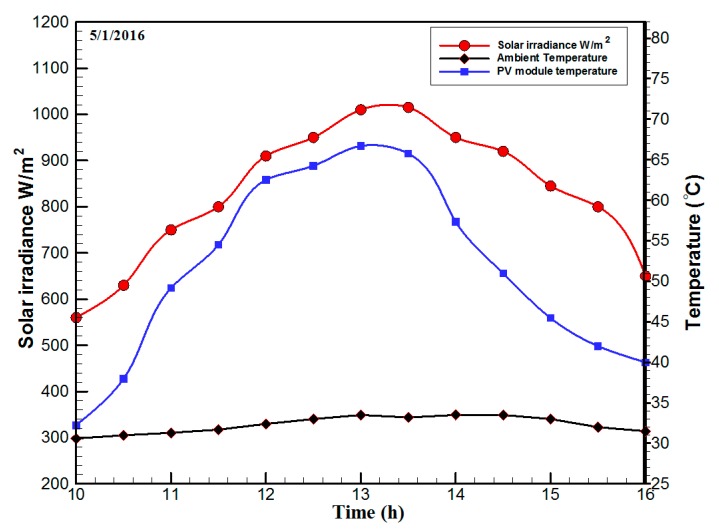
The hourly variation of solar irradiance intensity, PV temperature and ambient temperature of Universiti Kebangsaan Malaysia (UKM), Bangi on 5 January 2016.

**Figure 6:**
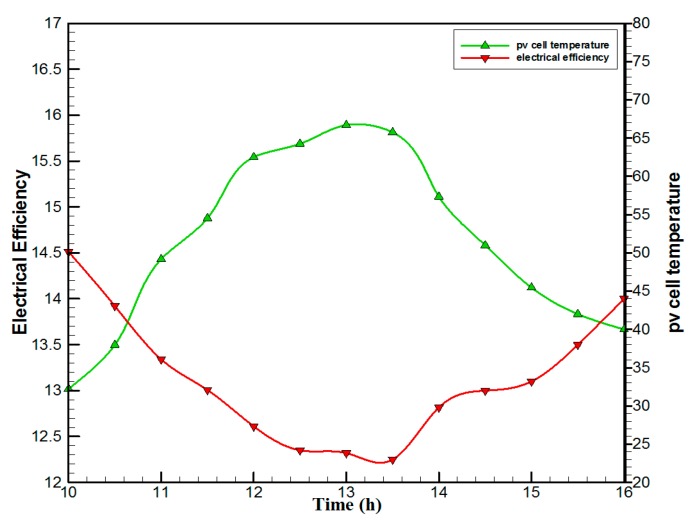
The hourly variation of electrical efficiency and solar cell temperature for the PVT solar collector with CPC.

The design parameters of the studied system are tabulated in Table 1. The hourly variations of the ambient temperature and solar intensity for 5 January 2016 are taken in Universiti Kebangsaan Malaysia (UKM), Bangi. Figure 5 shows that the solar intensity increased from 580 W/m^2^ at 10.00 a.m., to 1030 W/m^2^ at 1:30 p.m., then decreases to 540 W/m^2^ at 4:00 p.m. The ambient temperature increased from 30.5 ℃ at 10.00 a.m. to 33.8 ℃ at 1:00 p.m., then decreased to 31.5 ℃ at 4:00 p.m. The hourly variations of electrical efficiency and cell temperature of the PVT solar collector are shown in Figure 6. The electrical efficiency is inversely proportional to solar cell temperature. The solar cell temperature is at a maximum and electrical efficiency is at a minimum between 12:30–13:30 h. It is clearly seen that the electrical efficiency decreased from 14.5% at 10.00 a.m., to 12.25% at 1:30 p.m., then increased to 14% at 4:00 p.m. The PV cell temperature is increased from 32.5 ℃ at 10.00 a.m., to 66.5 ℃ at 13:00 p.m., and then decreased to 40 ℃ at 4:00 p.m.

**Figure 7:**
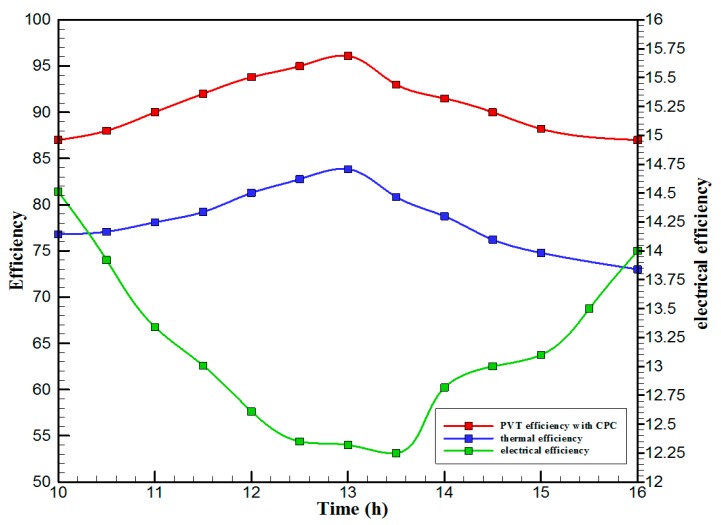
The hourly variation of electrical, thermal and PVT efficiency for the PVT solar collector with CPC.

**Figure 8:**
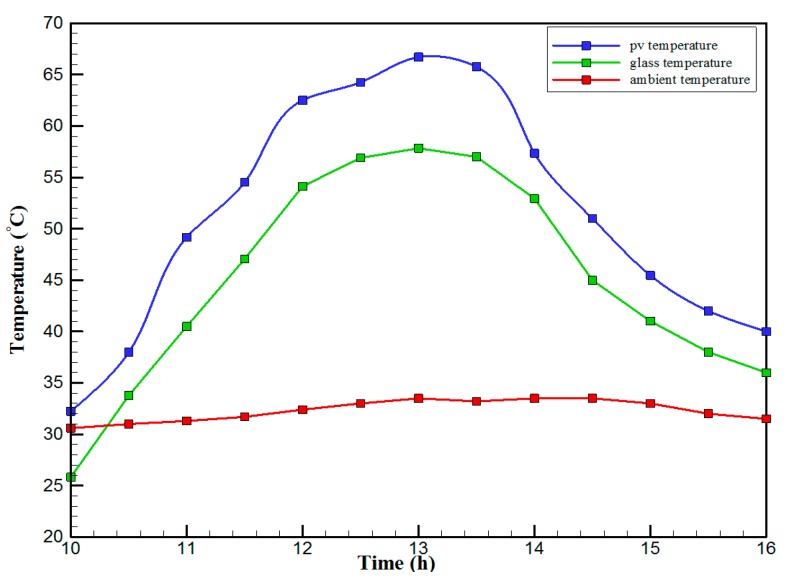
The hourly variation of PV, glass and ambient temperature for the PVT solar collector with CPC.

**Figure 9:**
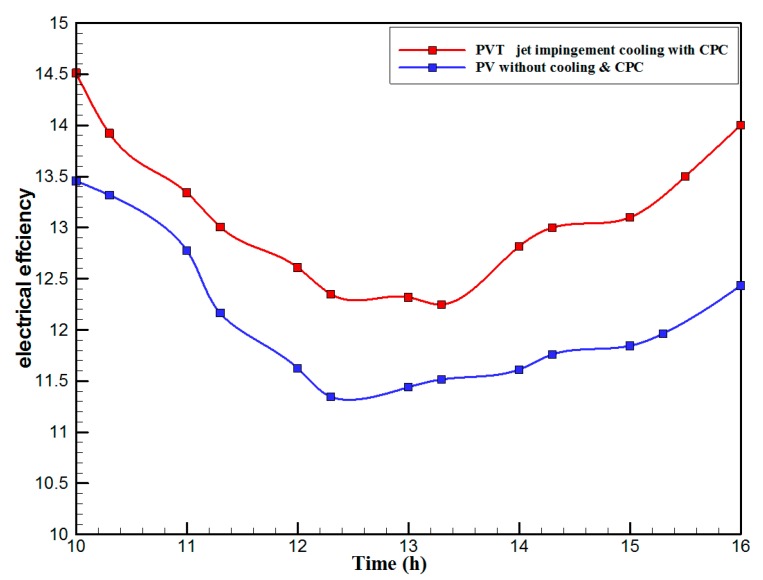
The hourly variation of electrical efficiency for the PVT solar collector with CPC and electrical efficiency for the PV module without cooling and CPC.

**Figure 10:**
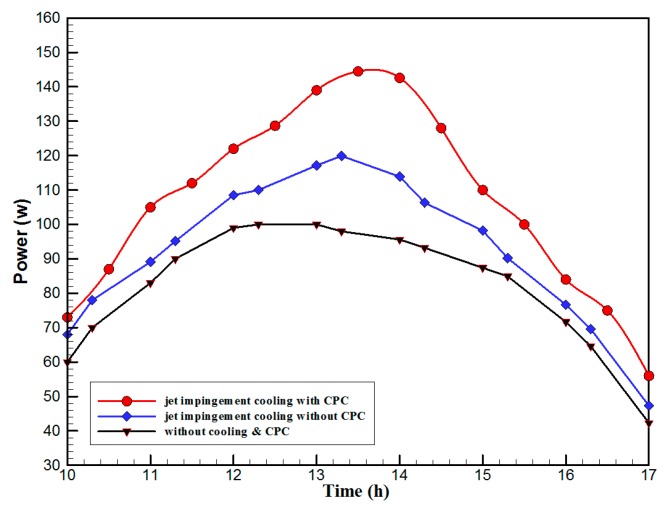
The hourly variation of electrical output power for the PVT-CPC collector, PVT without CPC and the PV module.

**Figure 11:**
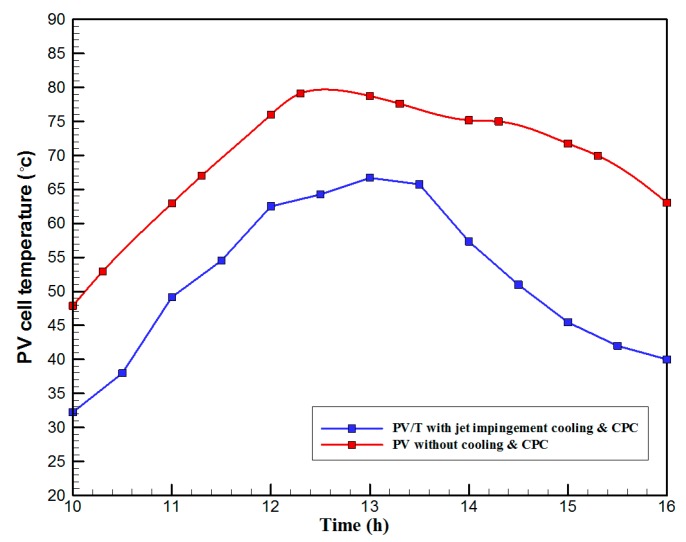
The hourly variation of the PV cell temperature for the PVT solar collector with CPC and the PV module without cooling and CPC.

**Figure 12:**
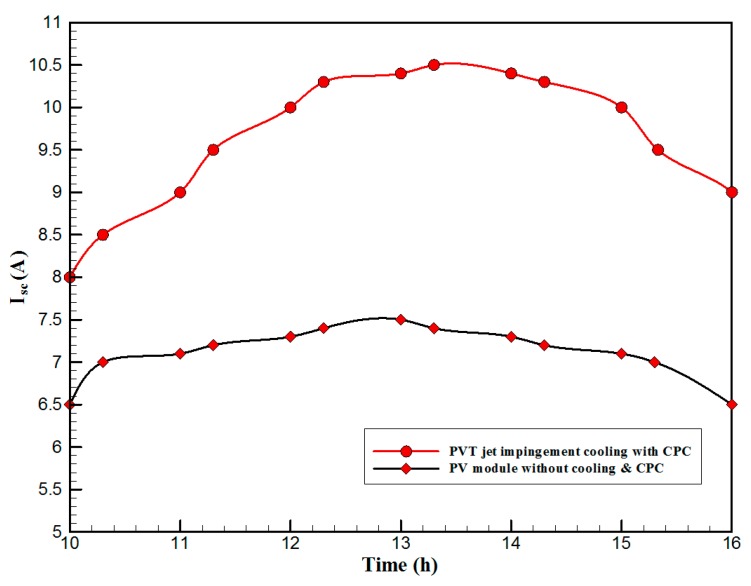
The hourly variation of short-circuit current for the PVT solar collector with CPC.

**Figure 13:**
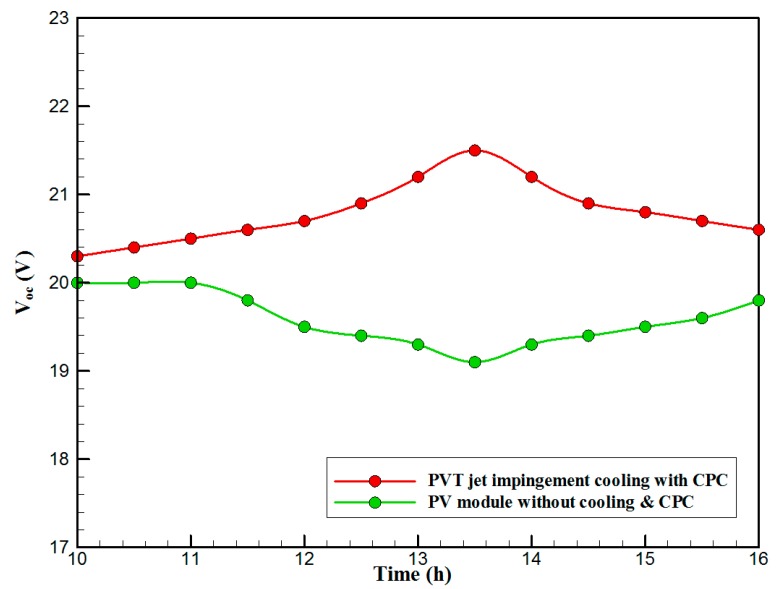
The hourly variation of open-circuit voltage for the PVT solar collector with CPC.

**Figure 14:**
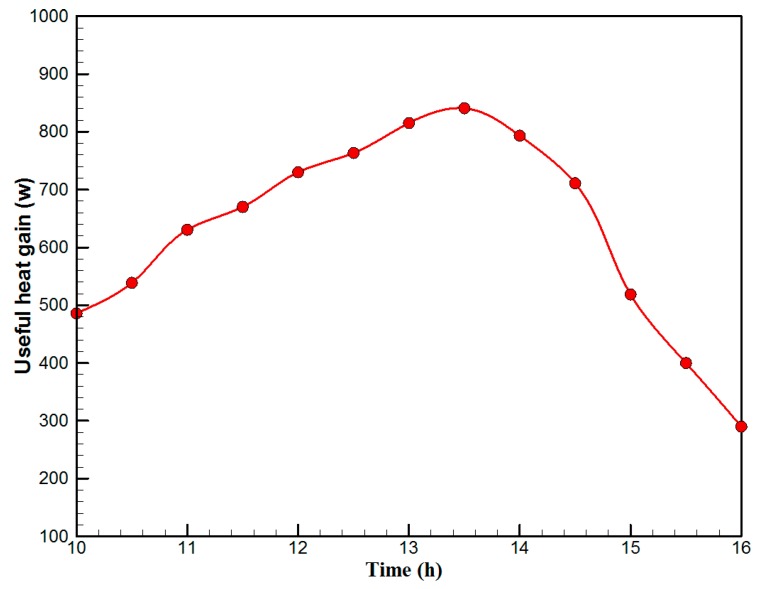
The hourly variation of useful heat gain for the PVT solar collector with CPC.

**Figure 15:**
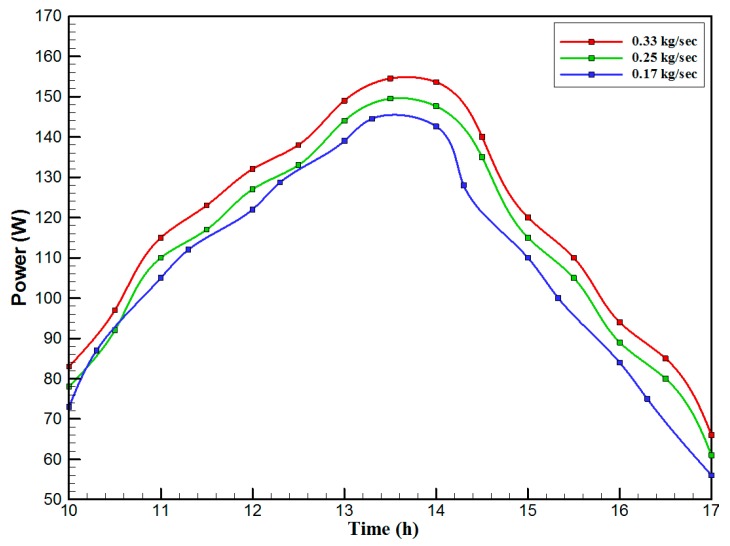
The hourly variation of output power for the PVT solar collector with CPC for different mass flow rates of water.

**Figure 16:**
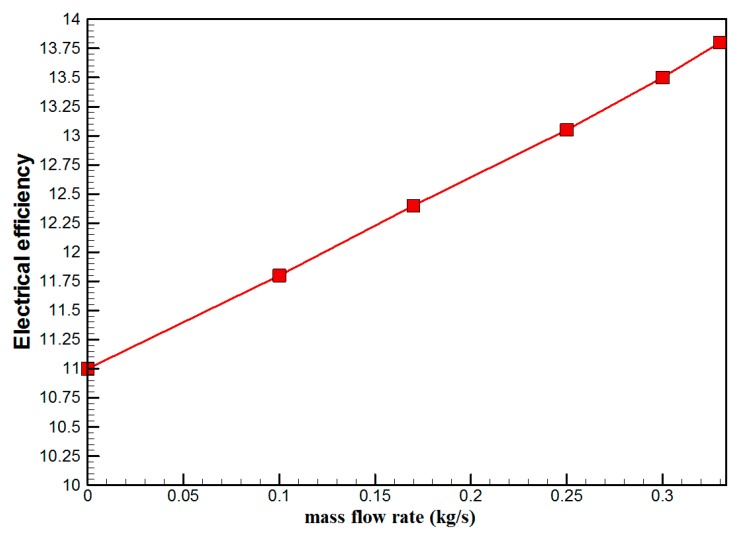
The variation of electrical efficiency with mass flow rate of water for the PVT solar collector with CPC.

**Figure 17:**
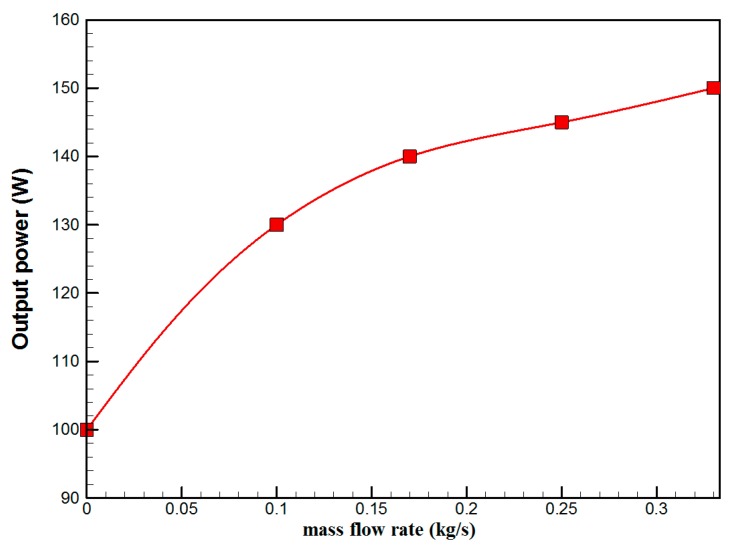
The variation of power output with mass flow rate of water for the PVT solar collector with CPC.

**Figure 18:**
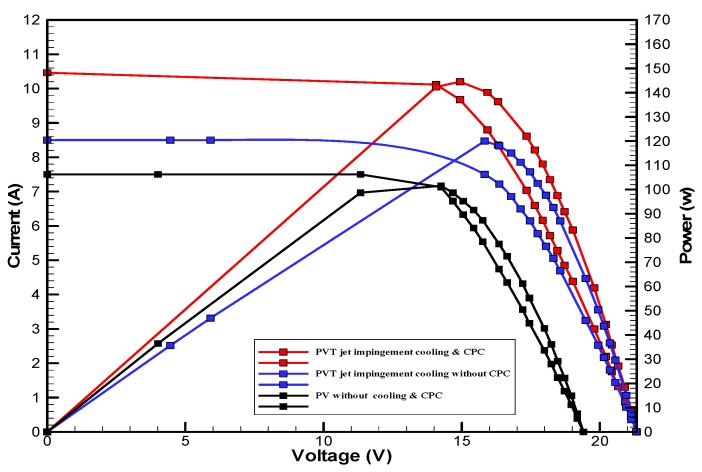
The I-V-P characteristic curve of the PV module in compared systems; PVT with CPC, PVT without CPC and PV free without CPC at Solar radiation 1000 w/m^2^.

Figure 7 shows the hourly variation of electrical, thermal, and PVT efficiency for the PVT solar collector with CPC. It can be seen that the electrical efficiency decreased from 14.5% at 10.00 a.m., to 12.25% at 1:30 p.m., and then increased to 14% at 4:00 p.m. The electrical efficiency decreased due to the increase of PV cell temperature from 32.5 ℃ at 10.00 a.m. to 66.5 ℃ at 13:00 p.m. It can also be seen that the thermal efficiency decreased from 84% at 10.00 a.m. to 81.5% at 1.00 p.m., then to 80% at 4:00 p.m. The overall efficiency decreased from 96% at 10.00 a.m., to 93% at 13:00 p.m., and then to 94% at 4:00 p.m. The hourly variations of PV cell temperature, glass temperature of PVT solar collector, and ambient temperature are shown in Figure 8. The results show that the solar cell temperature increased from 32 ℃ at 10.00 a.m., to 66.5 ℃ at 13:00 p.m., and then decreased to 40 ℃ at 4:00 p.m. The glass temperature of the PVT solar collector increased from 26 ℃ at 10.00 a.m., to 58 ℃ at 1:00 p.m., and then decreased to 36 ℃ at 4:00 p.m. The ambient temperature increased from 31 ℃ at 10.00 a.m., to 32.5 ℃ at 1:00 p.m., and then decreased to 31 ℃ at 4:00 p.m. The hourly variations of the electrical efficiency of the PVT-CPC solar collector and the PV module are shown in Figure 9. The results show that the electrical efficiency of the PVT-CPC exceeds that of the electrical efficiency of the PV module without CPC and jet impingement cooling all day. The use of CPC and cooling cell temperature via jet impingement lead to improved electrical efficiency for the PVT-CPC. The minimum electrical efficiency for the PVT-CPC and the PV module is noted between 12:30 to 13:30 p.m., due to increased cell temperature. It can be seen that the electrical efficiency for PVT with jet impingement decreased from 14.5% at 10.00 a.m. to 12.25% at 1:30 p.m., then increased to 14% at 4:00 p.m. It is also noted that the electrical efficiency of the PV module without cooling decreased from 13.5% at 10.00 a.m. to 11.4% at 12:30 p.m., then increased to 12.4% at 4:00 p.m. The electrical efficiency improved by 7% by using CPC and jet impingement cooling in the PVT solar collector at 1:00 p.m. The hourly variations of the output power of the PVT-CPC solar collector, the PVT solar collector without CPC and the PV module are shown in Figure 10. The results show that the usage of CPC with cooling the PV solar cell by jet impingement improve the power production of the PVT-CPC solar collector because the CPC can increase the solar intensity while the jet impingement reduces the PV temperature. It can also be seen that the output power of PVT without CPC exceeds the output power of the PV module. The Figure 11 show that the use of jet impingement cooling and CPC reflectors has a significant effect on the output power at solar noontime. It is clear that the electrical power output for PVT with CPC and jet impingement cooling increased from 72 W at 10.00 a.m. to 150 W at 1:30 p.m., and then decreased to 56 W at 4:00 p.m. The electrical power output for PVT with jet impingement cooling without CPC increased from 68 W at 10.00 a.m. to 120 W at 1:30 p.m., and then decreased to 48 W at 4:00 p.m. The electrical power output for the PV module without CPC and jet impingement cooling increased from 60 W at 10.00 a.m. to 98 W at 1:30 p.m., and then decreased to 42 W at 4:00 p.m. As a result of this, it can be clearly seen that the power output at 1:30 p.m. improved by 36% when using jet impingement cooling with CPC, and 20% when using jet impingement cooling without CPC in the PV module. It has been observed that the PV cell temperature for the PV module exceeds that of the solar cell temperature for the PVT at all times. The solar cell temperature was at a maximum between 12:00–14:00 h. The PV cell temperature for PVT with jet impingement and CPC increased from 32.5 ℃ at 10.00 a.m. to 67 ℃ at 1:00 p.m., and then decreased to 40 ℃ at 4:00 p.m. The PV cell temperature for the PV module without jet impingement and CPC increased from 48 ℃ at 10.00 a.m. to 80 ℃ at 12:30 p.m., and then decreased to 64 ℃ at 4:00 p.m. The hourly variation of short-circuit current for the PVT solar collector with CPC display is shown in Figure 12. It has been noted that the Short-Circuit Current for the PVT solar collector increases with increased solar radiation. The short-circuit current is at a maximum between 12:00 to 14:00 h. The short-circuit current for PVT with jet impingement and CPC increased from 8 A at 10.00 a.m., to 10.4 A at 1:00 p.m., then decreased to 9 A at 4:00 p.m. The short-circuit current for the PV module without jet impingement and CPC increased from 6.5 A at 10.00 a.m. to 7.5 A at 1:00 p.m., and then decreased to 6.5 A at 4:00 p.m. It can be seen that the short-circuit current improved by 28.5% using jet impingement cooling and CPC. The hourly variation of open-circuit voltage for the PVT solar collector with CPC is presented in Figure 13. The open-circuit voltage for PVT with jet impingement and CPC increased from 20.3 V at 10.00 a.m. to 21.5 V at 1:30 p.m., and then decreased to 20.5 V at 4:00 p.m. The open-circuit voltage for the PV module without jet impingement and CPC decreased from 20 V at 10.00 a.m. to 19 V at 1:30 p.m., and then increased to 19.8 V at 4:00 p.m. As a result of this, it can be seen that the open-circuit voltage improved by 11% when using jet impingement cooling and CPC. The hourly variation of useful heat gain for the PVT solar collector with CPC is shown in Figure 12. It is noted that the useful heat gain for the PVT solar collector is directly proportional to solar radiation is shown in Figure 14. The useful heat gain is at a maximum between 12:00–14:00 h. It is noted that the useful heat gain increased from 480 W at 10.00 a.m. to 840 W at 1:30 p.m., then decreased to 280 W at 4:00 p.m. The influence of multiple mass flow rate of water on the power output of the PVT-CPC solar collector is shown in Figure 15. It is noted that the power output of the PVT-CPC solar collector is directly proportional to the mass flow rate of water. The power output of the solar cell is enhanced with the cooling solar cell and reduced solar cell temperature. The electrical power output for the PVT-CPC increased from 83 W at 10.00 a.m. to 155 W at 1:30 p.m., and then decreased to 68 W at 4:00 p.m. when the mass flow rate was 0.33 kg/s. Figure 16 shows the variation of electrical efficiency with mass flow rate of water for the PVT solar collector with jet impingement cooling and CPC. Electrical efficiency is directly correlated to the mass flow rate. Figure 17 shows the variation of power output with mass flow rate of water for the PVT solar collector with jet impingement cooling and CPC. It is noted that the power output is directly proportional to the mass flow rate. The I–V–P characteristic plot of the PV module in the compared systems, the PVT solar collector with CPC at 67 ℃, PVT without CPC at 79 ℃ and the PV module without CPC at a solar radiation of 1000 W/m^2^ and temperatures are shown in Figure 18. The result shows the short-circuit current $I_{sc}$ of the PV module being improved by ~28% when using jet impingement cooling with CPC, and an improvement of 11.7% when using jet impingement cooling without CPC. The output power of the PV module is improved by 31% when using jet impingement cooling with CPC, and 16% without CPC.

## 6. Conclusions

In this study, an experimental investigation of a photovoltaic thermal water collector system with jet impingement cooling and CPC was presented. The electrical performance of the PVT system with and without CPC was compared to a simple PV module. A polycrystalline silicon solar module with a jet impingent cooling system, combined with a stainless mirror CPC was designed, assembled and analyzed. Experiments were carried out to analyze the influence of jet impingement of water in PVT-CPC on both the thermal and electrical performance. The experimental results showed that the integration of CPC in photovoltaic thermal collectors is superior in terms of electrical and thermal performance compared to a conventional flat plate PVT collector. The electrical and thermal efficiency of the PVT-CPC system increased while using a jet impingement cooling system due to the high transfer between the back of the PV cell and the cooling fluid. The results clearly show the increased mass flow rate leading to increased output power of the PVT-CPC system due to the cooled PV cells. The installation of the CPC increased the solar radiation of the PV, which increased output power. The electrical efficiency and power output are directly proportional to the mass flow rate. The result clearly showed that the electrical efficiency was improved by 7% when using CPC and jet impingement cooling in the PVT solar collector at 1:00 p.m. It was also evident that the power output improved by 36% when using jet impingement cooling with CPC, and 20% without CPC in the PV module at 1:30 p.m. The short-circuit current $I_{sc}$ of the PV module was improved by ~28% when using jet impingement cooling with CPC, and 11.7% without CPC. The output power of the PV module is improved by 31% when using jet impingement cooling with CPC, and 16% without CPC.

## Figures and Tables

**Table 1:** Specification sheet of the photovoltaic (PV) module.

Characteristic	Value
Cell type	Polycrystalline silicon
Number of cells	36 cells
Maximum power ($P_{max}$)	135 W
Open circuit voltage ($V_{oc}$)	21.80 V
Short circuit current ($I_{sc}$)	7.97 A
Maximum power voltage ($V_{mp}$)	18.12 V
Maximum power current ($I_{mp}$)	7.45 A
Cell open circuit voltage	0.6 V
Module efficiency (at STC)	15%
Cell size	156 × 156 mm^2^

**Table 2:** PVT solar collector characteristics.

Description	Symbol	Value	Unit
Ambient temperature	$T_{a}$	33	℃
Collector area	$A_{c}$	1	m^2^
Number of glass cover	N	1	–
Emittance of glass	$\varepsilon_{g}$	0.88	–
Emittance of plate	$\varepsilon_{p}$	0.95	–
Collector tilt	*θ*	0	°
Fluid thermal conductivity	$k_{f}$	0.613	W/m ℃
Specific heat of working fluid	$C_{p}$	4180	J/kg ℃
Back insulation conductivity	$k_{b}$	0.045	W/m ℃
Back insulation thickness	$l_{b}$	0.05	m
Insulation conductivity	$k_{e}$	0.045	W/m ℃
Edge insulation thickness	$l_{e}$	0.025	m
Absorber conductivity	$k_{abs}$	51	W/m ℃
Absorber thickness	$l_{abs}$	0.002	m
Transmittance	τ	0.88	–
Absorbance	α	0.95	–

**Table 3:** The design parameters of the PVT collector.

Parameters	Value
The length of PV module, L	2 m
The width of PV module, W	0.6 m
The wind velocity, v	2 m/s
The mass flow rate of water, $\dot{m}$	0.17 kg/s
The heat conductivity of glass cover	0.7 W/m K
The absorptivity of glass cover, $a_{g}$	0.05
The number of nozzles	36 pcs
The diameter of nozzle, d	0.001 m
The thickness of solar cell, $d_{c}$	0.0003 m
The heat conductivity of solar cell, $k_{c}$	148 W/m K
The spacing between nozzles and solar cell, H	1.0 mm

## Nomenclature

$A_{c}$	frontal area solar collector (m^2^)
*b*	collector width (m)
$C_{P}$	specific heat of working fluid (J/kg ℃)
d	diameter of nozzle (m)
$D_{h}$	hydraulic diameter (m)
$F^{\prime }$	collector efficiency factor
$f_{i}$	inlet fluid
$F_{R}$	heat removal efficiency factor
$G_{T}$	solar radiation at NOCT (W/m^2^)
$h_{fi}$	heat transfer coefficient of fluid (W/m^2^ ℃)
k	thermal conductivity (W/m ºC)
$I_{sc}$	Short-circuit current
L	tube length (m)
l	thickness (m)
$\dot{m}$	mass flow rate (kg/s)
N	number of glass cover
p	collector perimeter (m)
$Q_{u}$	actual useful heat gain (W)
G	solar radiation (W/m^2^)
T	temperature (℃)
$U_{L}$	overall heat transfer coefficient (W/m^2^ ℃)
$U_{t}$	top loss coefficient (W/m^2^ ℃)
v	wind velocity (m/s)
α	absorbance
θ	collector tilt
ϵ	emittance
τ	transmittance
η	efficiency
σ	Stefan-Boltzmann’s constant (W/m^2^ K^4^)
**Subscripts**	
a	ambient
abs	absorber thickness
c	cell
ele	electric
i	inlet
g	glass
j	jet
o	outlet
p	plate
m	mean plate
*PV*	photovoltaic
*PVT*	photovoltaic thermal
*r*	reference
th	thermal
w	wind
a	ambient
abs	absorber thickness
